# The Effect of GnRH Analogs on Body Mass Index in Girls with Central Precocious Puberty: A Single-Center Retrospective Study with a Literature Review

**DOI:** 10.3390/children12030336

**Published:** 2025-03-07

**Authors:** Ignazio Cammisa, Elena Malavolta, Federica Arzilli, Giulia Rotunno, Donato Rigante, Clelia Cipolla

**Affiliations:** 1 Department of Life Sciences and Public Health, Fondazione Policlinico Universitario A. Gemelli IRCCS, 00136 Rome, Italy; elena.malavolta01@icatt.it (E.M.); federica.arzilli01@icatt.it (F.A.); giulia.rotunno01@icatt.it (G.R.); donato.rigante@unicatt.it (D.R.); clelia.cipolla@policlinicogemelli.it (C.C.); 2 Department of Life Sciences and Public Health, Università Cattolica Sacro Cuore, 00168 Rome, Italy

**Keywords:** central precocious puberty, GnRH analog, body mass index, weight gain, metabolism, pediatrics, personalized medicine

## Abstract

**Background/Objectives:** Central precocious puberty (CPP) is defined by the premature onset of secondary sexual characteristics prior to the age of 8 and 9 years in girls and boys, respectively. The administration of GnRH analogs (GnRH-a) has become a cornerstone in the management of CPP, but effects on metabolic processes, particularly body mass index (BMI), remain a subject of ongoing investigation. This study aimed to investigate the relationship between GnRH-a treatment and BMI changes in a cohort of Italian children with CPP. **Methods:** We conducted a retrospective cohort study of 34 girls with idiopathic CPP, all treated with GnRH-a. Anthropometric parameters including BMI were collected at three time points: baseline, one year after treatment, and one year after treatment cessation. A comprehensive review of the medical literature concerning GnRH-a and BMI was performed. **Results:** Statistical analysis using the Wilcoxon and McNemar tests revealed a significant increase in BMI-for-age *z*-scores after one year of GnRH-a, with a slight increase also observed one year post-treatment of CPP. These findings suggest that GnRH-a treatment contributes to an increase in BMI, particularly in CPP children with a normal weight at baseline, although the overall impact on the progression of obesity remains minimal. A review of the existing literature supports the notion that changes in BMI during GnRH-a treatment are influenced by various factors, including baseline BMI, gender, and metabolic processes. **Conclusions:** Despite our findings suggesting the potential impact of GnRH-a on BMI, further longitudinal studies are necessary to fully understand the long-term metabolic consequences of GnRH-a therapy in children with CPP.

## 1. Introduction

Central precocious puberty (CPP) is defined as the onset of secondary sexual characteristics before the age of 8 in females and 9 in males and occurs due to the premature activation of gonadotropin-releasing hormone (GnRH)-secreting neurons [1,2]. The use of gonadotropin-releasing hormone analogs (GnRH-a) in the treatment of CPP has been a significant advancement in pediatric endocrinology, offering an active tool to delay onset of puberty in children who experience early sexual maturation [1,2]. GnRH-a work as inhibitors of the secretion of gonadotropins, thereby halting the premature activation of puberty and allowing the proper time for normal growth and development [2,3]. The primary effects of GnRH-a include reduction in growth velocity, regression or lack of progression of clinical pubertal signs, progressive decrease in the ratio of bone age (BA) to chronological age (CA), and increase in predicted adult height [3]. The efficacy and safety of GnRH-a has been established by many studies demonstrating their implementation in the clinical practice as a well-tolerated and safe treatment [1,2,3]. A growing body of research has started to investigate the impact of GnRH-a on various metabolic processes, including changes in body mass index (BMI), although the findings from existing studies remain somewhat inconsistent [4,5,6,7,8]. Some evidence suggests that GnRH-a treatment in children with CPP may lead to an increase in BMI, while other studies report no significant effect or even a reduction in body weight [4,5,6,7,8]. The mechanisms underlying the relationship between GnRH-a and metabolic changes, including variations in BMI, are complex and multifactorial. GnRH-a induce a state of hypogonadism by suppressing gonadal sex hormone production, particularly estrogen and testosterone. This hormonal disruption can lead to alterations in several metabolic processes, including appetite regulation, energy expenditure, and fat distribution [5,7,9]. Fat mass continues to accumulate in children with CPP undergoing treatment, while lean mass decreases due to a shortened prepubertal growth period and the “menopausal effect” of treatment. This effect is more pronounced in girls than in boys [9]. Furthermore, these children may have reduced actual energy expenditure, likely resulting from the loss of lean body mass and changes in appetite-regulating hormones. Furthermore, the effects of GnRH-a on BMI may be modulated by a variety of additional factors, such as the age at the initiation of treatment, the duration of therapy, and child’s baseline BMI and growth trajectory. These factors may interact in ways that influence the overall metabolic response to GnRH-a, contributing to the observed variability in outcomes between individuals [10,11,12]. Understanding how GnRH-a affect BMI is crucial, as it can have significant implications for patient management and long-term health outcomes, particularly in populations undergoing long-term treatment.

The primary aim of our study was to analyze the anthropometric characteristics of a cohort of Italian patients with CPP and to assess the potential correlation between GnRH-a treatment and BMI. Specifically, we investigated the BMI *z*-score at the time of diagnosis, after one year of treatment, and one year following treatment interruption. Additionally, a comprehensive review of the medical literature regarding GnRH-a and BMI was conducted, without restrictions on publication dates.

## 2. Materials and Methods

This was a single-center observational retrospective study that included children with idiopathic CPP treated with GnRH-a, all managed through a regular follow-up at the Pediatric Endocrinology Day Hospital of the Fondazione Policlinico Universitario A. Gemelli IRCCS, Rome. The inclusion criteria were as follows: (1) children with pubertal onset, meaning before 8 years of age in girls and 9 years of age in boys; (2) children with suspected CPP, confirmed by a pediatric endocrinology assessment and based on pubertal signs such as the onset of breast development and/or menses in girls and testis volume ≥ 4 mL in boys, an accelerated growth rate, or the advancement of bone age 1 year above the chronological age; (3) children with a diagnosis of CPP confirmed by a GnRH stimulation test, with a standard maximal dose of 100 µg (2.5 µg/kg), showing a luteinizing hormone (LH) peak > 5 IU/L; (4) children with CPP who were undergoing GnRH-a treatment (triptorelin 3.75 mg intramuscularly every 4 weeks). Basal LH, basal follicle-stimulating hormone (FSH), peak LH, and FSH after GnRH stimulation were measured by a chemiluminescence immunoassay (CLIA) according to our laboratory guidelines. Pituitary magnetic resonance imaging (MRI) was performed in all cases to exclude organic CPP. The exclusion criteria included the following: (1) children with conditions that could affect BMI, such as Cushing’s syndrome, growth hormone deficiency, hypothyroidism, or hypothalamic obesity; (2) children receiving medications that might influence BMI; (3) children diagnosed with organic CPP due to conditions such as brain tumors, head trauma, central nervous system infections, or radiation therapy targeting the head.

This screening process enabled us to select 34 female patients with confirmed CPP under GnRH-a treatment (Triptorelin administered intramuscularly once every 28 days, with recommended doses of 3.75 mg for subjects weighing >20 kg and 1.875 mg for subjects weighing ≤20 kg). Our study only includes female children in our center. All 34 patients presented to our clinic due to thelarche, with 10 out of 34 also exhibiting changes in body odor. None of the patients had a family history of CPP. The youngest patient included in the study was a girl diagnosed with CPP at the age of 1 year. Clinical suspicion arose due to the onset of thelarche and a pelvic ultrasound, performed to evaluate an associated congenital uropathy with hydronephrosis, which revealed a uterus larger than expected for the patient’s age. Subsequent clinical and diagnostic assessments, including the GnRH stimulation test, confirmed this diagnosis.

Specifically, we retrospectively analyzed their clinical records from January 2018 to December 2024. Follow-up at our center included the measurement of auxological parameters: weight, height, and BMI. Height was measured to the nearest centimeter using a stadiometer, and body weight was measured to the nearest 0.1 kg using a calibrated digital scale. BMI was calculated as weight (kg) divided by the square of height (m^2^). For each parameter, we calculated the respective *z*-score for age and sex to obtain homogeneous and comparable data, ensuring the comparability of measurements across children of different ages. This was carried out prior to the initiation of treatment for CPP, after one year of treatment, and one year after stopping treatment. We also assessed pubertal changes (progression or regression) during treatment. At the time of screening, only 19 children had GnRH-a treatment interrupted, with an average duration of 2.78 years (1.5–4.1 years). According to guidelines, the criteria for discontinuing therapy were as follows: (1) average a chronological age between 11 and 12 years in females; (2) an average bone age between 11 and 12 years in females; (3) a significant decrease in growth velocity (GV) during therapy (growth velocity dropping to 1.5–2 cm/year). We discontinued the treatment based on criteria 1 or 3.

No side effects were reported during GnRH-a therapy [2].

## 3. Ethical Approval

Ethics committee approval was not required, as the General Authorization to Process Personal Data for Scientific Research Purposes (Authorization No. 9/2014) specifies that retrospective archival studies utilizing coded data, which prevent the direct identification of subjects, do not necessitate formal ethics approval. However, all analyses and measurements conducted on our patients were in compliance with the standards of good clinical practice and the routine management of patients with CPP. The parents of all patients were informed about the objectives of the study, and after being contacted they provided written informed consent for participation.

## 4. Data Extraction

Data on anthropometric parameters, listed in Table 1, were recorded in an electronic database for subsequent statistical analysis.

## 5. Data Interpretation

We collected laboratory and anthropometric data from 34 female patients diagnosed with CPP. The mean age at the time of diagnosis was 6.9 years, with a range from 1 to 7.9 years. The acquired data included age, height, weight, BMI, and the corresponding *z*-scores for all patients at baseline (time 0) and one year after the initiation of GnRH-a. For 19 patients, these anthropometric parameters were available one year after the conclusion of GnRH-a. Regarding BMI-for-age *z*-scores, based on WHO definitions, children under 5 years of age were classified as follows:

- Overweight: BMI-for-age greater than 2 standard deviations above the WHO Child Growth Standards median.
- Obese: BMI-for-age greater than 3 standard deviations above the WHO Child Growth Standards median.

Children aged over 5 years were classified as follows:

- Overweight: BMI-for-age greater than 1 standard deviation above the WHO Growth Reference median.
- Obese: BMI-for-age greater than 2 standard deviations above the WHO Growth Reference median.

Children were classified as underweight if their BMI-for-age was below −2 standard deviations.

## 6. Statistical Analysis

Categorical variables were reported as numbers, while continuous variables were presented as means with standard deviations. To assess significant differences in BMI-for-age *z*-scores between the initial measurements (time 0) one year post-treatment and one year after treatment completion, we applied the non-parametric Wilcoxon test and the McNemar test. Statistical analyses were conducted using IBM SPSS Statistics software, version 25.0 (IBM Corporation, Armonk, NY, USA). A significance level of alpha < 0.05 was used to determine statistical significance in all analyses.

## 7. Results

**Figure 1:**
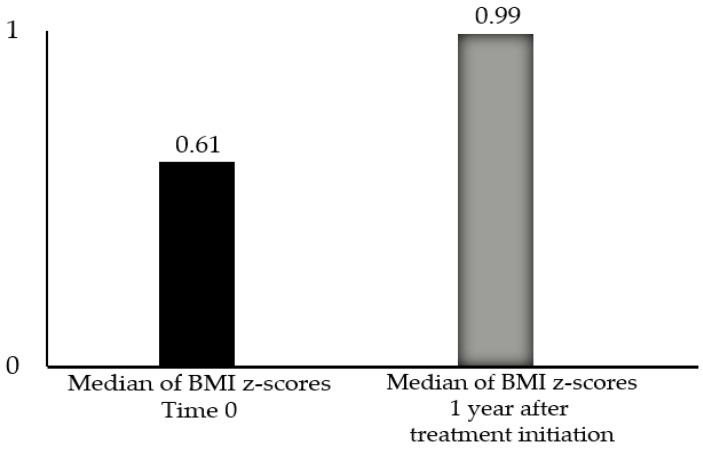
Differences in the medians of BMI-for-age *z*-scores at baseline (time 0) and 1 year after treatment with GnRH-a for patients with central precocious puberty; the Wilcoxon test revealed a statistically significant difference (*p* = 0.015).

**Figure 2:**
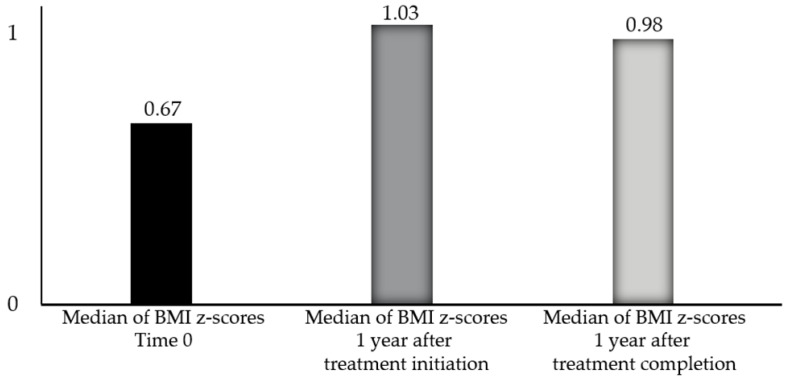
Differences in the medians of BMI-for-age *z*-scores at baseline (time 0), 1 year after treatment initiation (*p* = 0.19), and 1 year after treatment with GnRH-a (*p* = 0.035) in 19 patients with CPP whose anthropometric data were available one year after the end of treatment.

The non-parametric Wilcoxon test was conducted to assess whether BMI-for-age *z*-scores at baseline and 1 year after the initiation of treatment differed significantly. One year after the therapy’s initiation, the median BMI-for-age *z*-score was higher (0.99) compared to the baseline (0.61). The Wilcoxon test revealed a statistically significant difference between the two timepoints (*p* = 0.015) (Figure 1). For the 19 patients for whom anthropometric data were available one year after the end of treatment, the Wilcoxon test showed a statistically significant difference in BMI-for-age *z*-scores over time, with a higher median one year after treatment initiation (1.03) and one year after treatment completion (0.98) compared to the baseline (0.67) (respectively, *p* = 0.19 and *p* = 0.035) (Figure 2).

Regarding the classification, based on BMI-for-age *z*-scores, into normal weight, overweight, and obese categories, at the baseline (time 0), patients were distributed as follows: 20 were of a normal weight, 1 was underweight, 11 were overweight, and 2 were obese. One year after treatment, the distribution was as follows: 16 were of a normal weight, 1 was underweight, 15 were overweight, and 2 were obese. The McNemar test showed no statistically significant difference in the distribution of overweight subjects at the baseline and one year after treatment (*p* = 0.289). For the 19 patients for whom anthropometric data were available one year after the completion of therapy, the McNemar test did not show a significant difference in the distribution of overweight individuals (*p* = 0.688). Among these patients, 12 patients were classified as normal weight, 6 were overweight, and 1 was obese at baseline versus 10 of a normal weight, 8 overweight, and 1 obese one year after therapy.

## 8. Review of the Medical Literature

A review of the medical literature related to GnRH-a and BMI was conducted without restrictions on publication dates. Full-text English language records were included if they contained the terms “GnRH-a” and “BMI” or “weight”, along with “precocious puberty” and “BMI”. Two independent physicians (I.C. and E.M.) performed the literature search. Selected documents were cross-checked for accuracy (F.A. and C.C.), and duplicates were removed. The search resulted in the identification of 90 full-text manuscripts, of which 33 articles fully met the inclusion criteria, as reported in Table 2. The articles reviewed were published between 1999 and 2024, covering an overall 25-year period.

## 9. Discussion

GnRH-a are widely used in the treatment of various pediatric conditions, including CPP, where they are considered the standard of care: they exert their effects by binding to GnRH receptors, leading to the desensitization of pituitary gonadotrophins and the subsequent suppression of gonadal steroid secretion [2,3]. A variety of GnRH-a formulations are currently available, with the choice of formulations depending on either patient or physician preferences, as well as local regulatory approvals. Despite differences in routes of administration, dosing schedules, and the duration of action, these formulations have been shown to be equally efficacious [2,3]. While GnRH-a are highly effective in controlling the progression of puberty, their impact on metabolic processes, particularly on BMI, has significantly gained increasing attention in the scientific literature. Several studies have investigated this correlation, with some reporting an increase in BMI, while others observe no significant changes or even a reduction in body weight. This discrepancy in results is likely due to the complex relationship between GnRH-a treatment and BMI, which involves interactions between hormonal variations, different metabolic pathways, and individual factors. It is evident that GnRH-a primarily act through the suppression of gonadal hormones, such as estrogen and testosterone, playing a key role in regulating metabolism and resulting in changes in fat accumulation and lean body mass distribution [1,2,3]. Moreover, estrogen has been shown to reduce appetite by activating proopiomelanocortin (POMC) neurons via estrogen receptor α. It also regulates appetite-related hormones by enhancing leptin sensitivity, decreasing ghrelin activity, and increasing peptide YY levels [12]. Few clinical trials have validated changes in appetite-regulating hormones during GnRH-a treatment in children with CPP [38,39]. Palmert et al. evaluated daytime leptin levels over at least two years of GnRH-a treatment in 10 boys and 22 girls with CPP. They found that serum leptin levels increased in boys following the initiation of GnRH-a treatment, whereas no change was observed in leptin concentrations in girls [40]. On the other hand, Maffeis et al. reported a significant decrease in ghrelin concentrations during GnRH-a therapy in 20 girls with CPP [38]. These variations in neurotransmitter levels following GnRH-a treatment may have an impact on body weight in patients with CPP.

Our study was in line with other studies in the literature, demonstrating an increase in BMI after the initiation of GnRH-a therapy. Statistical analysis using the Wilcoxon test revealed significant changes in BMI-for-age *z*-scores over time. One year after the initiation of GnRH-a, the median BMI-for-age *z*-score significantly increased from 0.61 at baseline to 0.99, indicating an overall upward trend in BMI during treatment (*p* = 0.015). Similarly, one year after the conclusion of therapy, the median *z*-score further increased to 0.98, compared to 0.67 at baseline (*p* = 0.035), indicating continued weight gain in the post-treatment period.

Several studies have indicated that GnRH-a treatment can lead to increased BMI, particularly in girls [13,22,24,25,41]. Anik et al. observed significantly increased BMI during the first year of treatment (19.16 ± 2.8 vs. 20.7 ± 3.4), with the BMI standard deviation score (SDS) changing from 0.4 ± 0.8 to 0.8 ± 0.7 [22]. Similarly, Corripio et al. reported an increase in BMI-SDS of 0.43 ± 1.17 [24]. Vuralli et al. demonstrated an increase in BMI-SDS during GnRH-a treatment, chaing from 0.92 ± 0.74 to 1.20 ± 0.51, with a notable difference between normal weight children (0.45 ± 0.31 SDS) and overweight or obese children (0.03 ± 0.20 SDS) [5]. Arcari et al. documented an increase in baseline BMI-SDS from 1.1 ± 1 to 1.35 ± 0.95 at one year and 1.26 ± 0.1 at two years of treatment. However, normal weight patients showed a significant increase in BMI-SDS, rising from 0.3 ± 0.7 to 0.7 ± 0.8 at one year and 0.6 ± 0.8 at two years, while overweight patients showed a modest increase from the baseline (1.5 ± 0.2) to one year (1.7 ± 0.5). In contrast, obese patients did not show significant changes in BMI-SDS during treatment (baseline 2.4 ± 0.3, year one 2.4 ± 0.4, and year two 2.3 ± 0.6) [25]. Similarly, Yang et al. did not observe a significant overall change in BMI during GnRH-a treatment, but found that the normal weight group exhibited a significant increase in BMI-SDS, while the overweight group showed no change in BMI-SDS [11].

In our study, at baseline, the majority of the patients were classified as normal weight (20 patients), while 11 were overweight and 2 were obese. A small number (1 patient) was classified as underweight. After one year of GnRH-a therapy, there was a notable increase in the number of overweight patients, with 15 patients in this category compared to 11 at baseline. This could be attributed to the GnRH-a therapy, even though the number of obese patients remained unchanged, suggesting that this therapy did not have a significant impact on obesity status in this cohort. However, we did not evaluate other important factors, such as waist circumference, body composition, visceral fat, and above all dietary patterns and sedentary behaviors. Despite this, during follow-up, we consistently monitored weight and BMI, offering lifestyle recommendations when appropriate, including advice relating to a balanced diet and regular physical activity. When examining the classification of patients into normal weight, overweight, and obese categories, the McNemar test showed no significant changes in the distribution of these categories, either one year after treatment initiation or one year after treatment completion. This finding highlights that while the overall BMI-for-age *z*-scores improved, there was no substantial shift in the clinical classification of overweight or obese patients in the longer term, despite changes in their individual *z*-scores. These results suggest that baseline BMI-SDS may be an independent predictor of changes in BMI during GnRH-a treatment. The finding that changes in BMI-SDS during GnRH-a treatment depend on baseline weight status is not fully understood, although some explanations have been proposed, such as the influence of growth velocity and impact of weight management programs, including diet and exercise, recommended for overweight and obese children [4,5]. Regardless of baseline BMI, it appears that in children with CPP undergoing treatment, fat mass accumulation continues, as lean mass is reduced due to the shortened prepubertal growth period and the “menopausal effect” of the treatment itself. Additionally, these children may experience lower actual energy expenditure, likely resulting from a decrease in lean body mass [9]. The influence of pre-treatment BMI may be explained by the fact that, due to gonadal axis suppression, fat mass redistribution is more pronounced in lean children than in overweight or obese children. The pharmacological inhibition of the LH/FSH axis, which leads to the suppression of estrogen production, may contribute to the increased deposition of adipose tissue in lean children compared with those who are overweight or obese [36]. According to these findings, Loochi et al. documented changes in anthropometric and body composition measurements which indicated a gradual increase in adiposity and a decrease in muscle mass. The observed dynamics of body composition could be accounted for by neither participants’ self-reported dietary habits nor their physical activity levels and basal metabolic rate (BMR) [9]. Gender may also play a role in influencing the effect of GnRH-a treatment on BMI, as documented by Censani et al.: they demonstrated that females experienced a significant increase in BMI SDS (0.11 ± 0.36, *p* < 0.02) at 12 months, which was subsequently lost after 24 months. In contrast, males showed no significant changes in BMI SDS [31]. At the same time, Lim et al. found no BMI differences in 75 boys with CPP after 1 year and 2 years of therapy [32]. However, in most cases, after reaching adult height, BMI-SDS returned to baseline, suggesting that the effect of GnRH-a treatment on BMI may diminish as the child continues to grow [1]. The increase in BMI-SDS during GnRH-a treatment in normal weight children was reversible after treatment discontinuation, suggesting that GnRH-a do not result in the permanent elevation of BMI-SDS [5]. Lazar et al. conducted a retrospective study to compare treated and untreated CPP women, evaluating their BMI from diagnosis through to treatment cessation and into late adolescence and young adulthood; the authors found that the weight status of all women with a previous history of CPP, regardless of whether they had been treated, was similar to that of the general population despite their above-average BMI at the onset of puberty. Longitudinal analysis revealed an increase in BMI percentiles during GnRH-a treatment, followed by a decrease after treatment cessation, which continued into late adolescence and early adulthood [42]. Although our study indicated continued weight gain in the post-treatment period, suggesting that the therapy may have a lasting effect on the patients’ weight trajectories even after treatment cessation, a longer follow-up is needed to confirm these results.

On the other hand, some studies have shown that GnRH-a treatment had no apparent effect on BMI or BMI-SDS and did not exacerbate the prevalence of obesity during longer-term follow-ups [11,15,18,21,43,44]. Thus, GnRH-a treatment did not increase obesity, as patients with CPP maintained their baseline BMI-SDS during treatment, despite an overall increase in BMI following GnRH-a therapy [26]. For example, Pasquino et al. found that children treated with GnRH-a had BMI *z*-scores for chronological age of 0.39 ± 0.8 at the beginning of treatment, 0.41 ± 0.9 at the end of treatment, and 0.44 ± 1.0 during follow-up after treatment cessation. These differences were not statistically significant [15]. However, some studies demonstrated that, despite no change occurring in BMI-SDS during the GnRH-a treatment period, CPP patients experienced a two-fold increase in total fat mass compared to a normal population after reaching their final height post-treatment [45]. Interestingly, a few studies have reported a decrease in BMI or in body weight following GnRH-a treatment [14,46].

## 10. Limitations

Some limitations of this study must be declared: (1) it is a retrospective monocentric study, and its findings may not be generalizable to broader populations; (2) the sample size is small, and future studies should include a larger cohort with higher representation of male subjects; (3) we did not assess other relevant parameters, such as waist circumference, body composition, and visceral adiposity; (4) dietary habits and sedentary lifestyle factors were not investigated. These shortcomings could be addressed by conducting a proper prospective phase of the study. Therefore, longitudinal studies with larger sample sizes and long-term follow-up are needed to confirm our findings.

## 11. Conclusions

The effect of GnRH-a treatment on BMI during and after therapy continues to be a subject of ongoing discussion, primarily due to the diversity in the studies conducted so far. Differences in weight change can be linked to factors such as age, gender, and initial body weight. Furthermore, key factors influencing changes in BMI-SDS during GnRH-a treatment, such as genetics, diet, metabolic processes, and lifestyle, are often not considered and are challenging to control. A future perspective study could take these factors into consideration, eliminating them as potential bias. Our study, according to the scientific literature, indicates that GnRH-a treatment may lead to an increase in BMI, especially in children with CPP who have a normal weight at the start of treatment, although the overall effect on the progression of obesity is minimal. As a result, well-designed long-term controlled studies are necessary to properly determine the true impact of GnRH-a on the BMI of children with CPP and to gain a deeper understanding of its underlying mechanisms. Although the results are still preliminary, it would be advisable to include a surveillance protocol during follow-up in the guidelines to ensure the careful monitoring of BMI and weight gain, along with dietary advice and the promotion of regular physical activity and sports participation.

## Figures and Tables

**Table 1:** Anthropometric parameters of our population of patients with central precocious puberty.

Parameter	Mean (Max–Min)	N. of Subjects
Age time 0 (years)	6.9 (7.9–1)	34
Bone age 0 (years)	9.23 (2.4–12)	34
Bone age 1 year after therapy	9.83 (2.5–11.8)	12
Weight time 0 (kilograms)	27.2 (42.2–8.4)	34
Weight *z*-score time 0	0.6 (2.7–3.18)	34
Weight 1 year after therapy	33.5 (54–10.8)	34
Weight *z*-score 1 year after therapy	0.8 (3–3.58)	34
Weight 1 year after the end of therapy	47.29 (68.8–34)	19
Weight *z*-score 1 year after the end of therapy	0.93 (2.61–0.78)	19
Height time 0 (meters)	1.2 (1.4–0.75)	34
Height *z*-score time 0	0.52 (2.1–1.66)	34
Height 1 year after therapy (meters)	1.3 (1.5–0.87)	34
Height *z*-score 1 year after therapy	0.6 (2.45–2.20)	34
Height 1 year after the end of therapy (meters)	1.4 (1.5–1.3)	19
Height *z*-score 1 year after the end of therapy	0.4 (2.3–1.4)	19
BMI-for-age time 0 (kg/m^2^)	17.4 (23.3–13.9)	34
BMI-for-age *z*-score time 0	0.5 (2.4–2.19)	34
BMI-for-age 1 year after therapy (kg/m^2^)	19.0 (27.1–14.2)	34
BMI-for-age *z*-score 1 year after therapy	0.8 (2.95–2.59)	34
BMI-for-age 1 year after the end of therapy (kg/m^2^)	21.3 (29.3–16.3)	19
BMI-for-age *z*-score 1 year after the end of therapy	1 (2.3–0.2)	19

**Table 2:** Studies related to GnRH-a and BMI in children with idiopathic CPP.

Study	Country	Year	Design	Mean Age (Years)	Sample Size (M:F)	Intervention	Outcome
Mark R. Palmert et al. [6]	USA	1999	Prospective	Girls: 6.2 ± 0.2 Boys: 6.3 ± 0.8	110 (96:14)	Deslorelin 4–8 g/kg/day or Histrelin 10 mg/kg/day	The mean BMI SD score was 1.01 ± 0.1 for CA and for BA of 0.1 ± 0.1 at starting vs. 0.9 ± 0.1 and 0.6 ± 0.1 12–24 months after the interruption.
G. Chiumello et al. [13]	Italy	2000	Longitudinal	5.9 ± 1.9	16 (2:14)	Leuprolide or Triptorelin 3.75 mg/4 weeks	Increased fat mass compared to control group.
Teresa Arrigo et al. [14]	Italy	2004	Prospective	7.5 ± 0.9	101 (0:101)	Triptorelin 60 mg/kg/28 days	Average BMI-SDS and obesity prevalence significantly decreased at the end of therapy and during the period that followed therapy withdrawal.
A.L. Aguiar et al.	Brazil	2006	Retrospective	7.6 ± 0.1	176 (0:176)	Leuprorelin 3.75 mg/4 weeks or Goserelin 10.8 mg/12 weeks	BMI *z*-score increased from 1.5 ± 0.1 SD before treatment to 1.7 ± 0.2 SD after 24 months. In NW before treatment, this variation was greater (n = 112, 0.2 ± 0.1 SD, ρ = 0.01) than in those who were OW (n = 63, −0.9 ± 0.2 SD, ρ = 0.7).
Anna Maria Pasquino et al. [15]	Italy	2008	Retrospective	Treated: 6.5 ± 1.5 Untreated: 6.8 ± 1.6	119 (0:119)	Triptorelin 100–120 μg/kg/21–25 days	BMI SDS increased before, during, and after therapy.
Jung Hee Ko et al. [16]	Korea	2010	Prospective longitudinal	8.08 ± 1.0	121 (0:121)	Leuprolide acetate 100 g/kg/4 weeks	BMI not affected by GnRH-a.
Preamrudee Poomthavorn et al. [17]	Thailand	2011	Prospective	8.5 ± 1.0	58 (0:58)	Triptorelin acetate or Leuprolide acetate 3.75 mg/28 days	The BMI *z*-score returned to normal when they reached AH.
Seung Jae Lee et al. [18]	Korea	2012	Retrospective	7.93 ± 0.77	38 (0:38)	Leuprorelin acetate 30–90 μg/kg/28 days	BMI SDS was significantly increased at 12 months (0.79 ± 0.84, *p* = 0.049) and at 18 months (0.96 ± 0.83, *p* = 0.048).
Barbara Wolters et al. [4]	Germany	2012	Prospective	8	92 (11:81)	Triptorelin 3.75 mg/28 days	NW demonstrated a significant increase in BMI-SDS in the course of 1 year (+0.32 ± 0.66) in contrast to OW who showed a stable BMI-SDS (–0.02 ± 0.27).
Ana Colmenares et al. [19]	Venezuela	2014	Retrospective	Treated: 7.3 ± 1.5 Untreated: 7.7 ± 0.7	37 (0:37)	Triptorelin 3.75 mg/28 days	BMI *z*-score and OB/OW rates did not change significantly during 3 years of follow up. Weight *z*-scores were higher at 3 years in treated than in untreated girls.
Zohreg Karamizadeh et al. [20]	Iran	2014	Prospective longitudinal	8.5–12	30 (0:30)	Diphereline 80 mcg/kg/28 days	One year after the cessation of treatment: 73.3% had no change in BMI, 3.3% had decreased BMI and in 23.3% BMI had increased.
Kobra Shiasi Arani et al. [21]	Iran	2015	Prospective	7.46 ± 1.02	110 (0:110)	Triptorelin 3.75 mg/28 days	The BMI-SDS and OB was not significantly different at sixth and 12th months of treatment compared with baseline.
Ahmet Anik et al. [22]	Turkey	2015	Retrospective	8.5 ± 1.2	32 (0:32)	Leuprolide or Triptorelin 3.75 mg/28 days	BMI values showed statistically significant increase in the 1st year of treatment (19.16 ± 2.8 vs. 20.7 ± 3.4, *p* = 0.001).
Jessie N Zurita Cruz et al. [23]	Mexico	2016	Retrospective	6.8	121 (0:121)	Leuprolide 3.75 mg/month	BMI *z*-score increased from 0.87 to 1.32, and the rate of OW and OB increased from 40.5% to 70.3%.
Raquel Corripio et al. [24]	Spain	2016	Retrospective	7.33 ± 0.79	333 (0:333)	Triptorelin 80–120 μg/kg/month	BMI SDS increased by 0.43 ± 1.17 (95% CI: 0.20–0.64). At AH (n = 49), BMI-SDS was 1.51 ± 1.38, which was 0.60 ± 1.09 higher than at diagnosis.
Andrea J. Arcari et al. [25]	Argentina	2016	Retrospective	7.6 ± 1.3	117 (0:117)	Triptorelin acetate 100–120 μg/kg/28 days	NW showed a significant increase in BMI-SDS from 0.3 ± 0.7 to 0.7 ± 0.8 at one year of treatment and 0.6 ± 0.8 at two years (*p* < 0.001). In OW, a significant increase was only observed between baseline (1.5 ± 0.2) and one year of treatment (1.7 ± 0.5) (*p* < 0.05). OB girls showed no BMI-SDS changes during treatment
Sung Woo Kim et al. [26]	Korea	2017	Prospective longitudinal	8.49 ± 0.60	129 (0:129)	Triptorelin or Leuprolide acetate/28 days	BMI SDS increased significantly in the NW group after 2 years of GnRHa treatment.
Hae Sang Lee et al. [27]	Korea	2016	Retrospective	8.3 ± 0.6	383 (0:383)	Leuprolide acetate	Mean BMI SDS values after 2 years of treatment increased significantly only in NW (0.07 ± 0.69 vs. 0.25 ± 0.73, *p* < 0.001).
Jong Wan Yoon et al. [28]	Korea	2017	Retrospective	8.5 ± 0.5	127 (0:127)	GnRH-a 60–90 μg/kg/4 weeks	Increasing trend in the BMI-*z* score for BA was observed
Won Jun Yang et al. [11]	Korea	2017	Retrospective	8.6 ± 0.5	77 (0:77)	Not reported	The changes in BMI-SDS were significantly different between the NW and OW groups (0.15 ± 0.44 vs. −0.14 ± 0.40, *p* = 0.005)
Jina Park et al. [29]	Korea	2017	Retrospective	7.0–8.9	83 (0:83)	GnRH-a 60–90 mcg/kg/4 weeks	In NW subjects, BMI *z*-score was significantly increased during GnRHa treatment (−0.1 ± 0.7 vs. 0.1 ± 0.8, *p* < 0.001).
Pattharaphorn Sinthuprasith et al. [10]	Thailand	2019	Retrospective	8.27 ± 0.98	58 (1:57)	Leuprolide or Triptorelin 3.75 mg/4 weeks	BMI SDS at 1st and 2nd year of treatment were significantly increased in NW.
Andrea J. Arcari et al. [30]	Argentina	2019	Prospective longitudinal	7.8	19 (0:19)	Triptorelin acetate 100–120 μg/kg/28 days	There was an increase in BMI in NW but not in OW/OB patients.
Marisa Censani et al. [31]	USA	2019	Retrospective	8.49 ± 1.14	28 (3:25)	Leuprolide acetate or Histrelin acetate	Increased BMI SDS (0.11 ± 0.36, *p* < 0.02) at 12 months, which was subsequently lost by 24 months.
Kyung In Lim et al. [32]	Korea	2019	Retrospective	9.5 ± 0.5	75 (75:0)	Leuprorelin acetate or Triptorelin acetate/4 weeks	No significant changes in BMI from pretherapy values to the values after 1 and 2 years of GnRHa therapy (0.95 ± 0.75 vs. 0.94 ± 0.73, *p* = 0.667; 0.95 ± 0.75 vs. 0.98 ± 0.76, *p* = 0.532).
Dogus Vuralli et al. [5]	Turkey	2019	Retrospective	8.5 ± 1.0	138 (0:138)	Leuprolide acetate 3.75 mg/28 days	Changes in BMI-SDS (ΔBMI-SDS) during GnRHa differed between NW and OW/OB (0.45 ± 0.31 vs. 0.03 ± 0.20, *p* < 0.001). BMI-SDSs of both groups returned to baseline scores after 2 years.
Pınar Şimşek Onat et al. [33]	Turkey	2020	Retrospective	8.37 ± 0.97	54	Leuprolide acetate 3.75 mg/28 days	The number of OB and OW at diagnosis and the final assessment were similar (3.7% vs. 7.4% and 22.2% vs. 25.9%).
Young Suk Shim et al. [34]	Korea	2020	Prospective	9.5 ± 0.5	85 (85:0)	Leuprolide or Triptorelin 3.75 mg/4 weeks	Weight and BMI SDS did not change significantly.
Shiran Abargil Loochi et al. [9]	Israel	2021	Prospective observational	8.9 ± 0.9	32 (0:32)	Triptorelin 3.75 mg/4 weeks	Gradual increase in body fat accumulation and central obesity during the first year of treatment.
Z. Donbaloğlu et al. [35]	Turkey	2022	Retrospective	7.39 ± 0.76	43 (0:43)	Not reported	The rates of being OW and OW were increased (38.6% to 50% and 9% to 15.9%). At the end of the treatment, BMI SDS of the OW patients was still higher compared to the NW group.
Patrizia Bruzzi et al. [7]	Italy	2022	Retrospective	Group A: 7.86 ± 0.81 years Group B: 7.06 ± 1.61 years	57 (0:57)	Not reported	BMI SDS rose in the total population, which was attributable to the slight increase in the NW group. OW and OB girls did not show any significant changes.
Ana Luisa Leite et al. [36]	Portugal	2022	Retrospective	7.8 ± 1.6	92 (8:84)	Not reported	BMI-SDS increased with treatment but decreased a year after stopping GnRHa therapy (BMI-SDS 1.18 ± 1.0 at time I; 1.19 ± 1.0 at time II; and 1.06 ± 0.9 at time III; *p* = 0.06).
G. Tarçin et al. [37]	Turkey	2024	Prospective	7.6 ± 0.9	73 (0:73)	Leuprolide acetate 3.75 mg/28 days or 11.25 mg/3 months	Significant increase in body and truncal fat percentage.

AH (adult height), BA (bone age), BMI (body mass index), CA (chronological age), F (female), M (male), NW (normal weight), OW (overweight), OB (obese), SDS (standard deviation score).

## Data Availability

The original contributions presented in this study are included in the article. Further inquiries can be directed to the corresponding author.

## Abbreviations

Basal metabolic rate (BMR), body mass index (BMI), bone age (BA), central precocious puberty (CPP), chronological age (CA), chemiluminescence immunoassay (CLIA), follicle-stimulating hormone (FSH), gonadotropin-releasing hormone (GnRH), gonadotropin-releasing hormone analogs (GnRH-a), growth velocity (GV) luteinizing hormone (LH), magnetic resonance imaging (MRI), proopiomelanocortin (POMC), standard deviation score (SDS).
